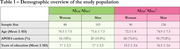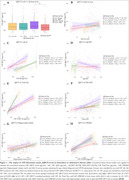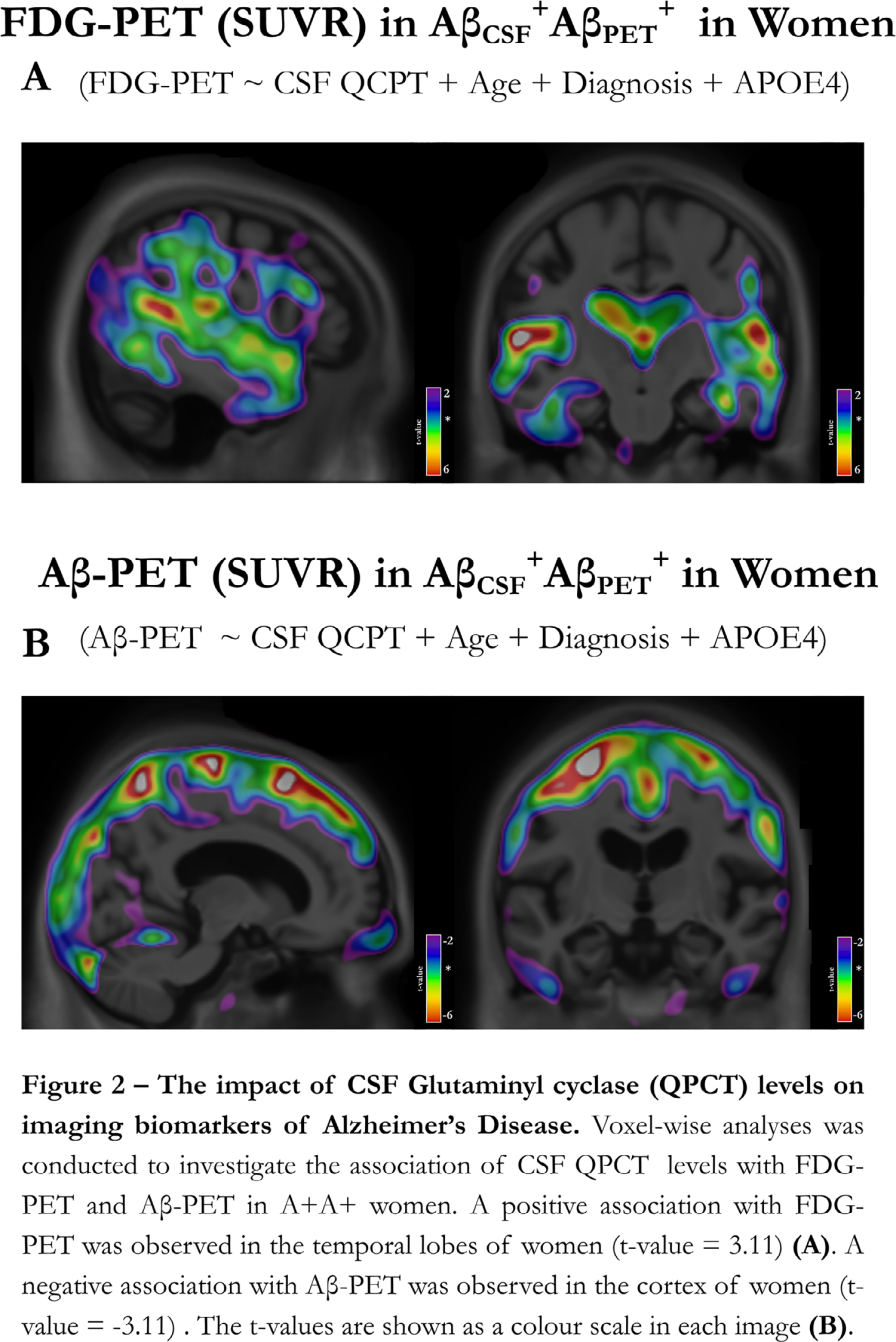# Sex‐specific associations between Glutaminyl Cyclase and Alzheimer's Disease biomarkers

**DOI:** 10.1002/alz70861_108965

**Published:** 2025-12-23

**Authors:** Marcelo Madrid de Bittencourt, Gabriela Mantovani Baldasso, Christian Limberger, Gabriel Lermen Hoffmeister, Débora Guerini de Souza, João Batista Teixeira da Rocha, Eduardo R. Zimmer

**Affiliations:** ^1^ Universidade Federal do Rio Grande do Sul, Porto Alegre, Rio Grande do Sul Brazil; ^2^ University of Cologne, Cologne, North Rhine‐Westphalia Germany; ^3^ Brain Institute of Rio Grande Do Sul, PUCRS, Porto Alegre, RS Brazil; ^4^ Universidade Federal de Santa Maria, Santa Maria, Rio Grande do Sul Brazil; ^5^ McGill Centre for Studies in Aging, Montreal, QC Canada

## Abstract

**Background:**

Glutaminyl‐peptide cyclotransferase (QPCT) catalyzes the formation of pyroglutamate‐modified amyloid‐beta (pGlu‐Aβ). pGlu‐Aβ is a neurotoxic and aggregation‐prone form of Aβ that accelerates plaque formation and contributes to Alzheimer's Disease (AD). While QPCT's role in promoting Aβ aggregation is well established, little is known about how its expression differs between sexes and associates with AD biomarkers. Given that women are more affected by AD, investigating sex‐specific associations between cerebrospinal fluid (CSF) QPCT levels and AD biomarkers could provide valuable insights into disease mechanisms.

**Method:**

Data were obtained from 405 individuals from the ADNI cohort at baseline, stratified by sex, CSF Aβ (AC) and Aβ‐PET (AP) positivity, which was defined based on the ratio *p* ‐Tau181/CSF Aβ42>0.028 and Aβ‐PET>1.11, respectively (Table 1). Generalized linear‐mixed models were applied to examine the association between CSF QPCT levels and AD biomarkers across groups (AC‐AP‐ women, AC‐AP‐ men, AC+AP+ women, AC+AP+ men), adjusting for age, clinical diagnosis and APOE phenotype. Additionally, voxel‐wise analyses investigated the association of CSF QPCT levels with FDG‐PET and Aβ‐PET.

**Results:**

CSF QPCT levels did not differ between AC‐AP‐ and AC+AP+. However, AC+AP+ women had higher CSF QPCT than AC+AP+ men. In AC‐AP‐, CSF QPCT correlated positively with CSF Aβ, Total‐Tau, and CSF sTREM2 in both sexes, while MoCA score only in women (Figure 1). In AC+AP+, CSF QPCT also correlated positively with Aβ42, Total‐Tau, and sTREM2 in both sexes; with hippocampal volume only in men and FDG‐PET only in women, primarily in temporal lobes as demonstrated in voxel‐wise analysis. In SUVr analysis, no associations were found between CSF QPCT and Aβ‐PET in either group. However, a negative association with Aβ‐PET was observed in the cortex of women at the voxel level (Figure 2).

**Conclusion:**

Our study demonstrates that AC+AP+ positive women had higher QPCT levels than AC+AP+ men. Additionally, stronger correlations between QPCT and imaging biomarkers, particularly FDG and Aβ‐PET, were found in women whereas men exhibited associations with hippocampal volume. These preliminary results suggest that, in AC+AP+ women, CSF QPCT protein levels are increased and associated with Aβ‐PET and FDG‐PET biomarkers.